# Genotyping and Phenotyping of Indigenous *Saccharomyces cerevisiae* from a New Zealand Organic Winery and Commercial Sources Using Inter-Delta and MALDI-TOF MS Typing

**DOI:** 10.3390/microorganisms12071299

**Published:** 2024-06-26

**Authors:** Junwen Zhang, Jeffrey E. Plowman, Bin Tian, Stefan Clerens, Stephen L. W. On

**Affiliations:** 1 Department of Wine, Food and Molecular Biosciences, Lincoln University, P.O. Box 85054, Lincoln 7674, New Zealand; cherie.zhang@lincoln.ac.nz (J.Z.); bin.tian@lincoln.ac.nz (B.T.); 2 Food and Bio-Based Products, AgResearch Ltd., Lincoln 7674, New Zealand; jeff.plowman@agresearch.co.nz (J.E.P.); stefan.clerens@agresearch.co.nz (S.C.); 3 Biomolecular Interaction Centre, University of Canterbury, Christchurch 8041, New Zealand; 4 Riddet Institute, Massey University, Palmerston North 4472, New Zealand

**Keywords:** MALDI-TOF MS, indigenous yeasts, *Saccharomyces cerevisiae*, commercial yeasts, inter-delta polymorphism, winemaking

## Abstract

We used inter-delta typing (IDT) and MALDI-TOF profiling to characterize the genetic and phenotypic diversity of 45 commercially available winemaking *Saccharomyces cerevisiae* strains and 60 isolates from an organic winemaker from Waipara, New Zealand, as a stratified approach for predicting the commercial potential of indigenous isolates. A total of 35 IDTs were identified from the commercial strains, with another 17 novel types defined among the Waipara isolates. IDT 3 was a common type among strains associated with champagne production, and the only type in commercial strains also observed in indigenous isolates. MALDI-TOF MS also demonstrated its potential in *S. cerevisiae* typing, particularly when the high-mass region (*m*/*z* 2000–20,000) was used, with most indigenous strains from each of two fermentation systems distinguished. Furthermore, the comparison between commercial strains and indigenous isolates assigned to IDT 3 revealed a correlation between the low-mass data (*m*/*z* 500–4000) analysis and the recommended use of commercial winemaking strains. Both IDT and MALDI-TOF analyses offer useful insights into the genotypic and phenotypic diversity of *S. cerevisiae*, with MALDI-TOF offering potential advantages for the prediction of applications for novel, locally isolated strains that may be valuable for product development and diversification.

## 1. Introduction

The changing nature of the wine industry globally is forcing wine producers to understand the demands of different markets better and provide superior and distinct wine styles accordingly. The choice of yeast species/strain is one efficient tool: complex volatile compounds produced by yeasts, including ethanol, esters, higher alcohols, sulfur-containing compounds, and many others, afford the wine distinctive attributes [1]. The concept of *terroir* has become a useful marketing tool to link essential wine quality attributes to the location of production [2]. The indigenous *Saccharomyces cerevisiae* strains, representing a source of natural biodiversity, can be different not only at the genomic level but also significantly in their metabolic profiles [3], yielding diverse organoleptic profiles unique to the regional characteristics of wines, demonstrating a better expression of *terroir* [4]. Moreover, the selected indigenous yeast strains may be better adapted to the local fermentation conditions than commercial strains [5,6,7] and preserve significantly more regional characteristics of the wine [8].

New Zealand *S. cerevisiae* is highly geographically structured at a local level, and this high diversity correlates with the European colonization of New Zealand, followed by subsequent diversification and admixture, and produces a distinct group of *S. cerevisiae* wine-group strains [9]. An investigation of the yeast population and diversity in the North Island of New Zealand depicted a region-specific sub-population and a reasonable level of gene flow of *S. cerevisiae* among different regions [10]. In addition, its regionally genetically differentiated *S. cerevisiae* population was reported to be associated with different wine phenotypes in terms of the ferment performance and chemical profiles [4]. However, there was no consistency found in intraspecies grouping by genotypic and phenotypic clustering [11]. However, the functional products of yeast gene expression (e.g., proteins) are likely to have a more direct correlation with the wine’s characteristics, and the strain-specific stress response [12] and the proteomic evolution in yeast adaptation to stressful winemaking conditions may correlate better to the unique properties of different oenological strains [13].

Earlier studies demonstrated the ability of matrix-assisted laser desorption ionization–time of flight mass spectrometry (MALDI-TOF MS) in yeast identification [14,15] and its potential in predicting the utility of *S. cerevisiae* strains of commercial benefit [16,17]. In brief, MALDI-TOF MS is a soft ionization technique, in which microbial cells are embedded in a suitable matrix that extracts and crystallizes native proteins, facilitating their ionization when exposed to a laser beam; the resulting ions are then accelerated through an electrostatic field and separated according to their *m*/*z* ratio until they reach the detector [18]. This technique is advantageous for its speed, cost-effectiveness, and ability to analyze complex mixtures of proteins with minimal sample preparation.

Furthermore, inter-delta typing (IDT) analysis is a well-established method for *S. cerevisiae* strain differentiation [19,20,21]. Inter-delta typing (IDT) is a molecular technique that can be used to differentiate yeast strains based on variations in the inter-delta regions of their genomes [21]. These regions exhibit polymorphisms that can be detected through PCR amplification, resulting in strain-specific banding patterns. Indeed, IDT has been claimed to be the most discriminative technique for the genetic property determination of wine yeast strains [3,8].

This study investigates the sequential use of IDT and MALDI-TOF analysis to determine the diversity of *S. cerevisiae* isolates of commercial and organic vineyard origins and to evaluate the potential for predicting the optimal applications of, especially, native yeast in wine production.

## 2. Materials and Methods

### 2.1. Yeast Strains

A collection of sixty *Saccharomyces cerevisiae* wild isolates and forty-five commercial strains were examined, and their genetic background and inter-delta types (IDTs) defined by their inter-delta profiles are noted in Supplementary Table S1. The *S. cerevisiae* isolates were purified from Pinot Noir (PN) grape must, obtained from an organic winery, Greystone Wines, Waipara, New Zealand [14]. Briefly, spontaneous fermentation was carried out in two different environments: (a) indoors in the winery and (b) outdoors in the vineyard. Yeasts were isolated from samples collected at four key stages of fermentation: (i) the first sign of fermentation, (ii) 6–8 °Brix drop, (iii) half of °Brix drop, and (iv) at the end of fermentation [14]. All strains were stored in YPD glycerol stock (30%, *v*/*v*) at −80 °C.

### 2.2. Inter-Delta Polymorphism Analysis

DNA was extracted using the Mag-Bind^®^ Environmental DNA 96 Kit (Omega, Bio-tek, Inc., Norcross, Georgia, USA) according to the protocol. The primer pair of delta12 (5′-TCAACAATGGAATCCCAAC-3′) and delta21 (5′-CATCTTAACACCGTATATGA-3′) used was recommended by Legras and Karst [21]. PCR amplifications were carried out in 25 μL reaction systems containing 10 × PCR buffer (Qiagen, Hilden, Cermany), 2.5 mM MgCl2 (Qiagen), 0.2 mM of each dNTP (Invitrogen, Carlsbad, CA, USA), 0.8 μM of each primer (Invitrogen), 2 U taq polymerase (Qiagen), and 2 μL DNA suspension. Amplification reactions were performed in Multigene Gradient (Labnet International, Inc., Edison, NJ, USA) with an initial denaturation at 95 °C for 4 min, followed by 35 cycles with a temperature profile of denaturation at 95 °C for 30 s, annealing at 50 °C for 30 s, and extension at 72 °C for 90 s, ending with a final extension period at 72 °C for 10 min and remaining at 4 °C.

The amplification products were separated by electrophoresis on 2% agarose gels subjected to 100 V for 90 min (PowerPac™ Basic, BIO-RAD, Hercules, CA, USA) in 1X Tris-borate-EDTA (TBE) buffer staining with 6% RedSafe™ Nucleic Acid Staining Solution and visualized under UV light. The digital images were acquired using Molecular Imager^®^ Gel Doc™ XR+ with Image Lab™ software (Version 6.0.0 build 25, BIO-RAD). The molecular mass marker 1 kb Plus DNA Ladder (Invitrogen) was used as the inter-gel control.

### 2.3. MALDI-TOF MS

#### 2.3.1. Sample Preparation

The sample preparation was as described previously [15]. In brief, yeast strains were cultured on YPD agar (Difco, Thermo Fisher Scientific, Waltham, MA, USA) for 3 days at 28 °C on 3 different days to obtain 3 biological replicates. Then, 1~3 colonies were picked using a sterile 200 μL pipette tip and emulsified into 300 μL deionized water. Afterwards, 900 μL absolute ethanol was added and vortexed for 1 min. After centrifugation (12,100× *g*, 4 min) (Eppendorf AG, Minispin 5452, Hamburg, Germany), the pellet was kept and air-dried in a laminar-flow hood. Prior to protein extraction, the samples were stored for up to 2 months at −20 ℃.

To extract proteins, 50 μL of 70% formic acid (*v*/*v*) was added to the yeast pellet and mixed thoroughly by vortexing for 1 min; then, 50 μL of acetonitrile (ACN) was mixed for the same time. The protein extract was obtained by centrifugation (12,100× *g*, 4 min). An equal volume of protein extract and α-cyano-4-hydroxycinnamic acid (HCCA) matrix solution (10 mg/mL in 75% ACN and 2.5% trifluoroacetic (TFA)) were mixed well, and 1 μL of this mixture was deposited onto the MALDI ground steel target plate (MTP 384, Bruker Daltonics^®^, Billerica, MA, USA) till dry. For technical replication, each extract was spotted onto three individual wells, therefore yielding 9 spectra per strain.

#### 2.3.2. Mass Spectra Acquisition

The MALDI-TOF mass spectra were automatically acquired on a Ultraflex III TOF/TOF MS instrument (Bruker Daltonics^®^, Bremen, Germany), operating in positive ion detection mode using a Smartbeam^TM^ laser at 200 Hz, pulsed-ion extraction time of 120 ns, and laser power 80%. The voltage of the ion source was set as 25.00 kV (ion source 1), 23.55 kV (ion source 2), and 6.01 kV (lens). The samples were analyzed using the linear detector in the high-mass range *m*/*z* 2000–20,000 and the reflector detector in the low-mass range *m*/*z* 500–4000. The final spectrum was an average accumulation of 2000 single spectra that were gathered. Each single spectrum was recorded from 10 random raster spots.

The mass spectrometer was externally calibrated in every experiment at regular intervals, using the calibrant position in the middle of each tetrad of spots. For the low-mass range *m*/*z* 500–4000, a peptide II standard (Bruker Daltonics^®^) (bradykinin 1–7 [M + H]^+^ at *m*/*z* 757.3992, angiotensin II [M + H]^+^ at *m*/*z* 1046.5418, angiotensin I [M + H]^+^ at *m*/*z* 1296.6848, substance P [M + H]^+^ at *m*/*z* 1347.7354, bombesin [M + H]^+^ at *m*/*z* 1619.8223, ACTH clip 1–17 [M + H]^+^ at *m*/*z* 2093.0862, ACTH clip 18–39 [M + H]^+^ at *m*/*z* 2465.1983, and somatostatin 28 [M + H]^+^ at *m*/*z* 3147.4710) was used. For the high-mass range *m*/*z* 2000–20,000, an in-house protein standard comprising insulin [M + H]^+^ at *m*/*z* 5734.52, cytochrome C [M + H]^+^ at 12,360.99 and [M + H]^2+^ at 6180.99, myoglobin [M + H]^+^ at 16,952.30 and [M + H]^2+^ at 8476.65), aprotinin [M + H] ^+^ *m*/*z* 6511.51, and β-lactoglobulin [M + H] ^+^ *m*/*z* 18,363.00 was used.

#### 2.3.3. Data Analysis

The raw mass spectra were exported in .txt format using FlexAnalysis software (version 3.0. Bruker Daltonics^®^) and imported into the software BioNumerics version 7.6 (Applied Maths). Spectral pre-processing was achieved at a default setting, but the rolling disc value for baseline subtraction was adjusted to 150. The Kaiser window value for smoothing and the signal to noise ratio (S/N) for peak filtering were adjusted according to the quality of spectra.

A composite profile of each strain was obtained using 9 spectra derived from three technical replicates of each of three biological replicates. A cluster analysis was performed using the Pearson correlation coefficient and the UPGMA (unweighted-pair group method with arithmetic mean) algorithm. MDS (multidimensional scaling)—available in BioNumerics version 7.6—was performed based on the similarity matrix calculated using the metric algorithm Pearson coefficient. In brief, MDS is a statistical technique used to analyze the similarity or dissimilarity of data by representing it as distances in a low-dimensional space [22]. MDS is advantageous for its ability to provide a visual representation of the relationships in high-dimensional data, making it easier to identify patterns, clusters, or outliers.

## 3. Results

### 3.1. Inter-Delta Polymorphism of Commercial Strains

**Figure 1:**
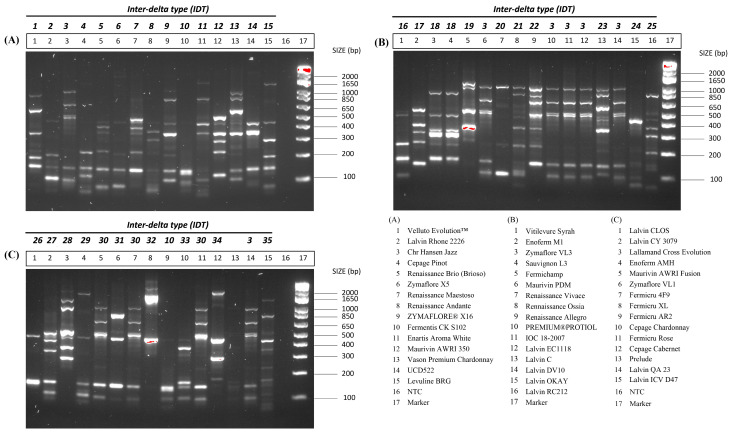
Inter-delta profiles of forty-five wine commercial strains. (**A**) Gel electrophoresis results for strains in the key A; (**B**) Gel electrophoresis results for a second set of strains listed in the key B; (**C**) Gel electrophoresis results for a third set of strains listed in the key C. DNA marker: 1 kb Plus DNA Ladder (Invitrogen). The number in bold and italics represents the inter-delta type, while the number in the box refers to the sample in the provided key in the bottom right corner. Please note that the red color observed in some bands is automatically produced when scanning the gel, indicating a high intensity of this band.

We observed thirty-five distinct inter-delta polymorphism patterns among the forty-five commercial wine strains studied (Figure 1). Both Fermentis CK S102 and Fermicru AR2 displayed identical patterns (IDT 10), as did Sauvignon L3 and Zymaflore VL3 (IDT 18). For PDM strains, typically recommended for Champagne production, two inter-delta types were identified: IDT 3 (present in Viniflora Jazz, Premium Protiol, Lalvin EC1118, IOC 182007, Maurivin PDM, Lalvin DV10, and Lalvin QA23) and IDT 30 (present in AWRI Fusion, Fermicru 4F9, and Fermicru Rose). These two types exhibited similar patterns with minor variations in band intensity at ~500 bp.

### 3.2. Inter-Delta Polymorphism of Wild Isolates

**Figure 2:**
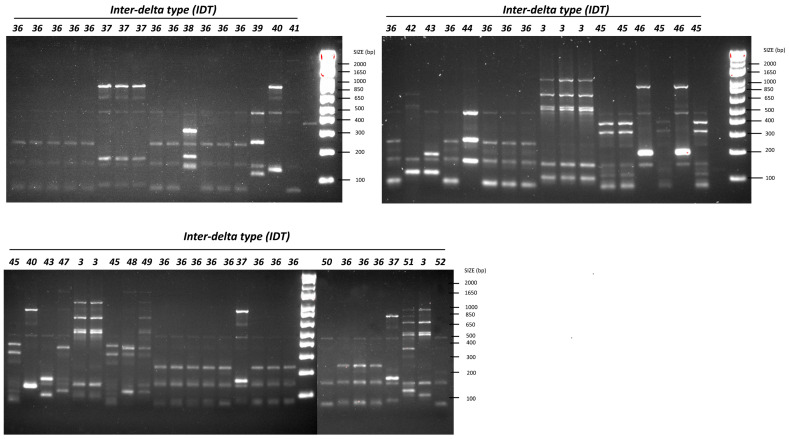
Inter-delta profiles of 60 *Saccharomyces cerevisiae* isolates. DNA marker: 1 kb Plus DNA Ladder (Invitrogen). The number in bold and italics represents the inter-delta type. Please note that the red color observed in some bands is automatically produced when scanning the gel, indicating a high intensity of this band.

An additional seventeen novel types were detected in the wild isolates, including one instance of IDT 3 in strains SV4–11, SV4–18, SV4–19, SW4–11, SW4–12, and SW4–13 (Figure 2). The types isolated during each fermentation stage in both vineyard and winery ferments are summarized in Table 1 and Table 2. The vineyard ferments had almost twice as many *S. cerevisiae* strains (fifteen) as the winery ferments (eight). Four common types (IDT 3, IDT 36, IDT 43, and IDT 45) were isolated in both ferments, with IDT 36 being dominant throughout the fermentation process. Strains unique to each ferment are indicated by an asterisk (*).

**Figure 3:**
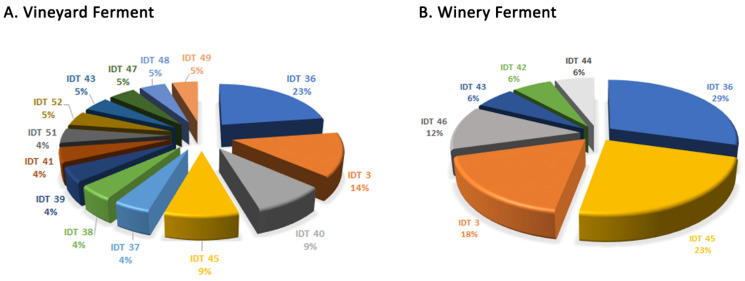
The percentage of each strain isolated at the final fermentation stage in (**A**) vineyard and (**B**) winery ferments. IDT: inter-delta type.

In the final fermentation stage, as depicted in Figure 3, we noted a pronounced variance within the *S. cerevisiae* community. Specifically, (i) the vineyard ferment was predominantly governed by IDT 36 (23%), IDT 3 (14%), IDT 40 (9%), and IDT 45 (9%); (ii) the winery ferment was mainly driven by IDT 36 (29%), IDT 45 (23%), IDT 3 (18%), and IDT 46 (12%). IDT 50 was only identified at the start of the vineyard fermentation, showing only minor differences from the prevalent IDT 36, notably lacking the band approximately at 220 bp.

### 3.3. MALDI Profile Analysis

The strains Fermicru AR2 and Safoeno^TM^ CK of IDT 10 generated protein profiles with 99% similarity. Similar observations were made for the IDT 18 groups (Sauvignon L3 and Zymaflore VL) and the IDT 46 groups (SW4_17 and SW4_19). Among the IDT 30 strains, Fermicru Rose and Fermicru 4F9 exhibited a 98% similarity, while AWRI Fusion was grouped into a distinct cluster, which could be due to its hybrid nature between *S. cerevisiae* and *S. cariocanus*.

**Figure 4:**
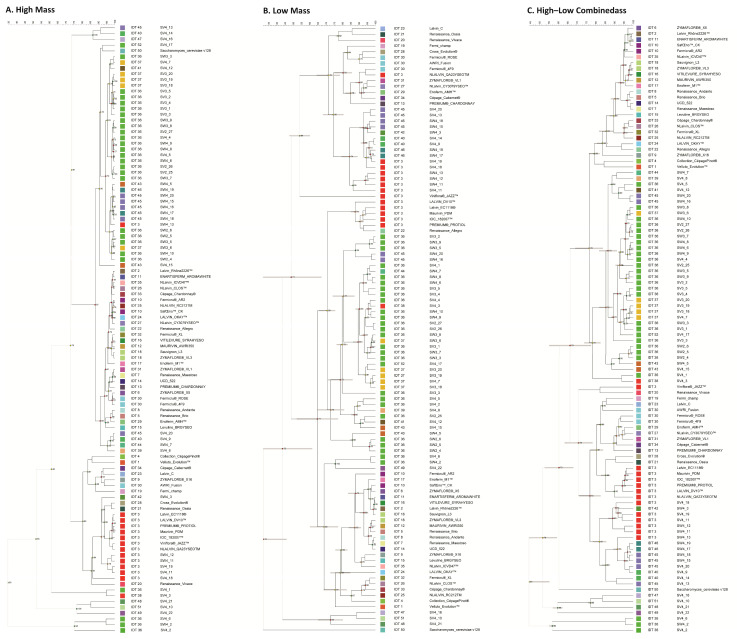
Cluster analysis of (**A**) high-mass profiles, (**B**) low-mass profiles, and (**C**) the combination of high- and low-mass profiles from forty-five commercial strains and sixty wild *Saccharomyces cerevisiae* isolates with the genetic information (inter-delta analysis) annotated, using the Pearson correlation coefficient and UPGMA algorithm. IDT: inter-delta type.

Commercial and wild strains belonging to IDT 3 were generally assigned to the same group, with the exception of SW4_13 (Figure 4A). Relative to the highly similar proteomic profiles (97%) derived from the commercial IDT 3 strains, the wild strains displayed greater diversity. The strains belonging to IDT 43 (SV4_15 and SW4_5) and IDT 40 (SV4_9 and SV4_14) displayed similarities of 92% and 81%, respectively. Furthermore, the similarity between their low-mass profiles was 86% for SV4_15 and SW4_5 and an impressive 99% for SV4_9 and SV4_14. It seems that the isolation source (vineyard and winery) has a non-negligible influence on the yielded protein profiles.

**Figure 5:**
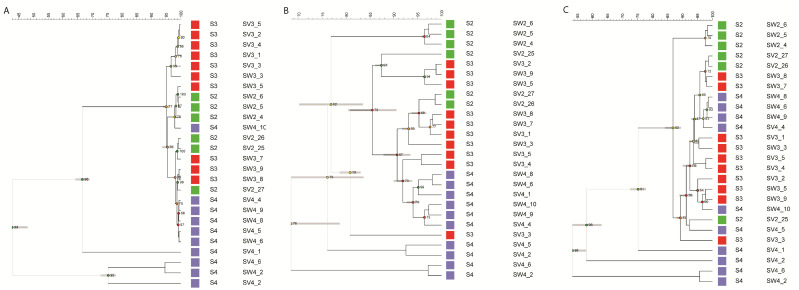
Cluster analysis of (**A**) high-mass profiles, (**B**) low-mass profiles, and (**C**) the combination of high- and low-mass profiles from IDT 36 using the Pearson correlation coefficient and UPGMA algorithm. S1: stage 1, S2: stage 2, S3: stage 3.

**Figure 6:**
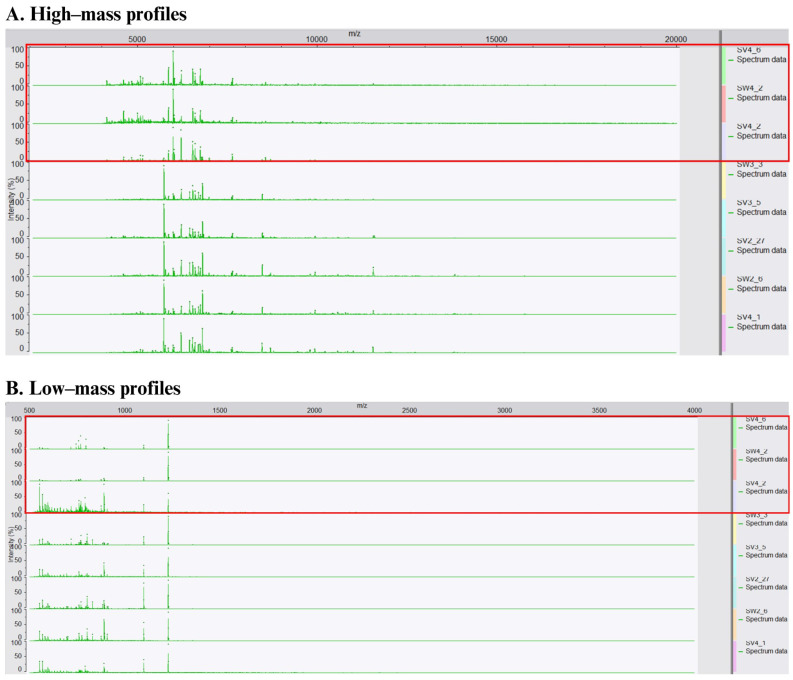
The comparison of profile patterns (**A**) high-mass and (**B**) low-mass profiles from eight isolates of strain 1 in three different clusters (SV4_6, SW4_2, SV4_2/SW3_3, SV3_5, SV2_27, SW2_6/SV4_1).

In the case of IDT 36 (Figure 5), there was a tendency for isolates from the same fermentation stage to group together. Within each fermentation stage, isolates from the same fermentation system (i.e., winery or vineyard) clustered closer. Excluding SV4_1, SV4_6, SV4_2, and SW4_2, all the high-mass profiles of IDT 36 were grouped in one cluster with a high similarity of 95%. Consistent with this, the protein profiles from SV4_6, SV4_2, and SW4_2 were visually distinct as displayed in Figure 6A, corroborating their position in the dendrogram. However, SV4_1 did not differ significantly from others. The same trend was observed in the low-mass profiles, suggesting a relationship between the high-mass and low-mass profiles. Furthermore, this relationship was quantified with a regression value of 58.45%, as computed through the Pearson correlation, thereby confirming their connection.

**Figure 7:**
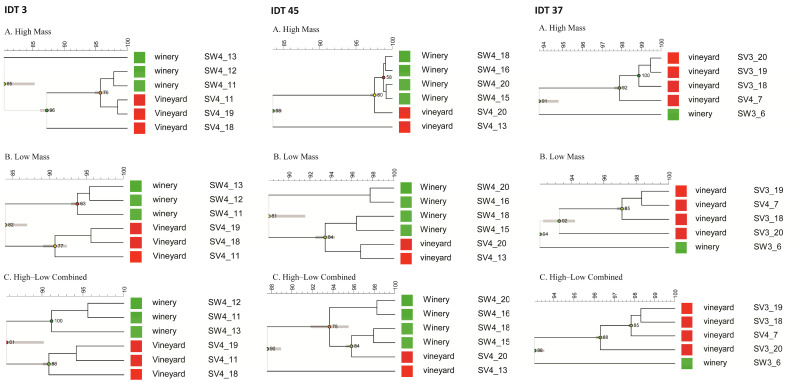
Cluster analysis of (**A**) high-mass profiles, (**B**) low-mass profiles, and (**C**) the combination of high- and low-mass profiles from IDT 3, IDT 45, and IDT 37 using the Pearson correlation coefficient and UPGMA algorithm.

A similar correlation of MALDI clusters with the isolation source was evident for IDT 3, IDT 45, and IDT 37 (Figure 7). IDT 3 and IDT 45 were exclusively isolated from the final stage of fermentation, while IDT 37 was mainly isolated from the third stage, with only one instance from the final stage. Without the interference of the fermentation stage, location appeared to be the most influential factor. Consequently, it can be inferred that isolates from similar environments display highly comparable protein profiles in both high- and low-mass moieties when they share the same genotypes.

**Figure 8:**
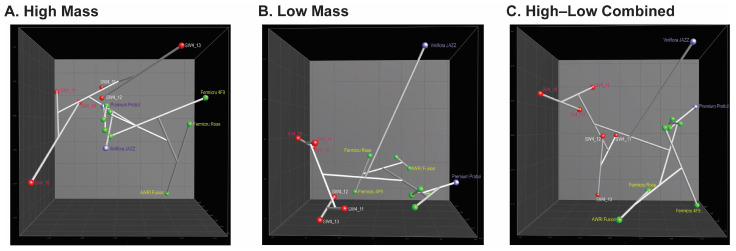
MDS analysis of (**A**) high-mass profiles, (**B**) low-mass profiles, and (**C**) the combination of high- and low-mass profiles derived from commercial and wild strains belonging to IDT 3 and IDT 30. Red: wild strains, green: commercial PDM strains, purple: commercial wine strains. Of the latter, Viniflora Jazz is recommended mainly for rose and red wine; Premium Protiol is particularly suited for white wine.

Cluster analyses of the low-mass profiles grouped differently to the high-mass profiles (Figure 4B), yet still indicated the same tendency for strains sharing the same inter-delta types to cluster closer. The similarity between Fermicru AR2 and Safoeno^TM^ CK (IDT 10) was 92%, that between Sauvignon L3 and Zymaflore VL (IDT 18) was 98%, and between SW4_17 and SW4_19 (IDT 46) it was 98%. Even though the strains belonging to IDT 3 were not in the same cluster, they were still closely located. Interestingly, PDM strains with different inter-delta types (IDT 30 and IDT 3) grouped closer by their low-mass profile than their high-mass profiles. This observation suggests that the derived low-mass data may be more indicative of their oenological properties (cf. Supplementary Table S1), while the high-mass data may better reflect the taxonomic relationship. This assumption was further corroborated by the MDS analysis (Figure 8).

Figure 8A clearly shows strains of AWRI Fusion, Fermicru 4F9, and Fermicru Rose (IDT 30) grouping separately from the others, consistent with the inter-delta typing result. Among the IDT 3 strains, the commercial strains were closer, whereas the isolates exhibited a higher variance. These results highlight the accuracy of the high-mass data in strain typing and indicate the influence of the isolation source on protein expression. For instance, the wild isolates were from Pinot Noir wild fermentation in New Zealand, while to the best of our knowledge, most commercial strains were isolated from Europe and varied grape varietals.

Our multidimensional scaling analysis (MDA, Figure 8B) showed that the distance between isolates was shortened in the low-mass profile, and the isolation sources—winery and vineyard—divided them into two minor branches, wild versus commercial strains. Notably, the strain Viniflora Jazz formed a unique branch distinct from the others. This could be attributed to its unique recommended application for rose and red wines. Hence, the combination of high-mass and low-mass profiles appears to offer a balance between the genotype and the environmental influence (or phenotypes) (Figure 8C).

## 4. Discussion

Our investigation revealed substantial genetic diversity within both commercial and indigenous yeast populations, consistent with findings from previous studies [3,19,23]. The relationships observed between the inter-delta polymorphism patterns and the MALDI profiles provided valuable insights into the relationship between genetic background, environment, and yeast characteristics.

Fermentations conducted at the winery and vineyard differed solely by location, with the vineyard fermentation being carried out outdoors at the grape growing site, potentially capturing a broader variety of indigenous *Saccharomyces cerevisiae* strains. This approach sought to amplify the current vintage’s influence on the final wine product. As expected, a marked difference was found in strain diversity between the vineyard ferment (fifteen types from thirty-four isolates) and winery ferment (eight types from twenty-six isolates), despite both originating from the same grape juice batch. This finding supports the current understanding of the influence of the microbial aspect of *terroir*, as suggested in our previous study concerning non-*Saccharomyces* yeasts [14], as well as the role of geographic location at global and regional scales, grape variety, and environmental conditions in shaping the genetic diversity and population structure of the *S. cerevisiae* wine group [9,24].

Despite being sourced from the same grape juice batch, only four of the eighteen types—IDTs 3, 36, 37, and 45—were found in both ferments. These accounted for 51% of vineyard and 76% of winery ferments, demonstrating the influence of the isolation source and fermentation stages on strain diversity and protein expression. A subset of three types of IDTs—IDTs 42, 44, and 46—were unique to the winery ferment, which are likely resident communities within the winery/cellar environment [25]. The occurrence of these specific *S. cerevisiae* strains at each winery supports their potential use in preserving the typicity of wines, even at the winery level [19]. Of the remaining strains, only one or two isolates were recovered, which, while not statistically significant, hold high industrial value. It is well established that indigenous yeast strains contribute to a greater expression of *terroir* fingerprint, and their utilization has indeed become an emerging trend in winemaking [26]. A singular type, IDT 3, was observed in both the isolates and the commercial strains. Given that *S. cerevisiae* yeast strains used for beer, bread, and wine production are genetically and phenotypically different from natural isolates [27], it is plausible that the isolates from IDT 3 might represent a commercial strain or a descendent thereof.

Further, we observed that genetically identical strains from different fermentation systems/stages displayed different protein profiles. In line with our previous findings [28], a specific strain can exhibit diverse MALDI protein profiles under different growth conditions. The changing and stressful environment during the alcoholic fermentation process did not affect the inter-delta polymorphism [20]. This observation underscores the relative stability of genotyping compared to phenotyping, hinting at the possible role of epigenetic influences. This was particularly evident in the analysis of IDTs 3, 36, 37, and 45 (Figure 5 and Figure 7). Epigenetic traits represent a stable heritable phenotype that arises from changes in a chromosome without alterations in the DNA sequence [29]. The epigenetic programs provide yeasts with phenotypic plasticity, enabling them to respond to fluctuating circumstances and thrive in niches [30]. Hence, the epigenetic inheritance might account for the differential protein profiles stemming from the same inter-delta types. Previous reports have indicated that the source of isolation exerts substantial influence on strain performance during the fermentation, with indigenous strains exhibiting superior adaptation to the ecological and technological features of their local winegrowing area [3]. This underlines the potential limitations of DNA-based techniques for yeast strain selection, suggesting that they may overlook strains with unique traits, as Ilieva, et al. [8] pointed out that similarity in the DNA profiles of strains does not necessarily translate into similar wines. Notably, recent studies show that dietary epigenetic compounds impact the wine’s chemical composition and sensory profile [31], further emphasizing the importance of understanding the environmental and epigenetic factors in organic winemaking which completely relies on the indigenous yeast community.

The application of MALDI-TOF MS in this study revealed interesting patterns in different mass ranges, pointing to a potential relationship between low-mass data and yeast metabolites, a key contributor to the aroma and flavor profiles of wine [32]. Our recent research also emphasizes the superior capacity of low-mass-range data (*m*/*z* 500–4000) in differentiating industrial strains (wine, beer, wild, and laboratory strains) [16]. In this study, the MDS analysis of IDT 3 strains further supported its value; the low-mass features seem to be more responsive to external stimuli compared to the genetic background. Compared to the high mass (Figure 8A), the isolates from same fermentation system clustered closer in low-mass plotting (Figure 8B). Among the commercial yeasts, the distance between PDM strains recommended for Champagne production and Premium Protiol was closer than that from Viniflora Jazz. This discrepancy is likely to be related to their technological properties and probably the original isolation source, as shown by the wild isolates. The ability to adapt metabolic activity in different environments seems to be an important factor, as it can affect the overall wine quality and potentially introduce novel secondary metabolites [3]. According to the manufacture instructions, Premium Protiol was selected to enhance the varietal and especially thiolic compounds in white wine because of its ability to express the thiolic compounds, while the outlier Viniflora Jazz was isolated from Shiraz and selected for its capability to achieve a fast and reliable alcoholic fermentation, primarily on rose and red wine. The other interesting aspect is the origin of those commercial stains; most of the tested strains were sourced from Europe, whereas Viniflora Jazz originated from South Africa. The high diversity of New Zealand *S. cerevisiae* strains was suggested to be correlated with European colonists, followed by subsequent diversification and admixture [9].

Welker [33] indicated that only those microorganisms capable of producing a high diversity of secondary metabolites can yield strain-specific mass patterns (*m*/*z* 500–2500). In light of this, the strains divided by low-mass data may express diversified secondary metabolites that contribute to a different wine quality. Thus, in order to select yeast strains with desirable properties, a series of assays need to be performed to determine such things as fermenting vigor, sugar consumption, glycerol production, resistance and sulfur dioxide production, resistance and alcohol production, low hydrogen sulfide production, and low volatile acidity production [19,34]. Compared to the tedious traditional yeast selection procedure, the application of MALDI-TOF MS demonstrates its potential as a rapid and easy-to-operate tool for indigenous yeast selection. Future work should focus on assessing the important oenological properties of the indigenous strains identified in this study, particularly the technological properties between groups of strains with identical inter-delta types.

## 5. Conclusions

In conclusion, our findings underscore the observation that MALDI profiles are an outcome of both genetic backgrounds and environmental influences. The data collected in this study indicate a strong correlation between genotypes and high-mass data and an innovative association between secondary metabolites and low-mass data. This study pioneers the use of the low-mass range (*m*/*z* 500–4000) in wine yeast analysis. Encouragingly, our current data promote the inclusion of low-mass data to create a more comprehensive MALDI-TOF MS database. The high-mass data are of significant taxonomic relevance, as suggested in previous research, whereas the low-mass data demonstrated superior performance in differentiating industrial strains. Therefore, the integration of both datasets allows for a balanced representation of genotype and phenotype relationships.

Significantly, beyond its capacity for identification, MALDI-TOF MS paves the way for groundbreaking yeast epigenetics studies, fostering innovation in fermented food and the selection of indigenous yeast. By streamlining the selection process, MALDI-TOF MS offers an efficient and cost-effective approach to identify novel strains as starter cultures for winemaking. This advance can greatly preserve the unique wine quality inherent to specific oenological areas, enhancing the competitiveness of the regional wine industry.

To complete the map of indigenous yeasts’ biodiversity, further work should be undertaken on the determination of technological properties and metabolic profiles during inoculate fermentation of the *Saccharomyces cerevisiae* isolates. Moreover, it would be intriguing to encompass different vintages or winemaking regions in further studies, given the effect of *terroir* in yeast diversity. All in all, continuing research on MALDI-TOF MS could revolutionize the wine industry, with the possibility that it could be extended to the whole fermented food industry. The application and development of this technology could ultimately enhance the quality, specificity, and competitiveness of fermented food products, ushering in a new era of food innovation and diversity.

## Figures and Tables

**Table 1:** *Saccharomyces cerevisiae* strains isolated from four key stages of fermentation in vineyard (V) and winery (W) wine production systems.

SV1 [1]	SV2 [3]	SV3 [8]	SV4 [22]	SW2 [3]	SW3 [6]	SW4 [17]
IDT 50 * (1)	IDT 36 (3)	IDT 36 (5)	IDT 36 (5)	IDT 36 (3)	IDT 36 (5)	IDT 36 (5)
		IDT 37 (3)	IDT 3 (3)		IDT 37 (1)	IDT 45 (4)
			IDT 45 (2)			IDT 3 (3)
			IDT 37 (1)			IDT 46 * (2)
			IDT 38 * (1)			IDT 42 * (1)
			IDT 39 * (1)			IDT 43 (1)
			IDT 40 * (2)			IDT 44 * (1)
			IDT 41 * (1)			
			IDT 43 (1)			
			IDT 47 * (1)			
			IDT 48 * (1)			
			IDT 49 * (1)			
			IDT 51 * (1)			
			IDT 52 * (1)			

Note: The number of *Saccharomyces cerevisiae* isolates examined at each stage is given in square brackets, and the number of each strain is given in round brackets. The * is used to indicate the specific strains observed in each ferment system. IDT: inter-delta type, SV1: 1st fermentation stage from vineyard sample, SV2: 2nd fermentation stage from vineyard sample, SV3: 3rd fermentation stage from vineyard sample, SV4: 4th fermentation stage from vineyard sample, SW2: 2nd fermentation stage from winery sample, SW3: 3rd fermentation stage from winery sample, SW4: 4th fermentation stage from winery sample.

**Table 2:** The number and isolation source of each strain from Pinot Noir wild fermentation.

No.	Strain	Number of Isolates	Source
1	IDT 36	26	Winery/Vineyard
2	IDT 3	6	Winery/Vineyard
3	IDT 45	6	Winery/Vineyard
4	IDT 37	5	Winery/Vineyard
5	IDT 40	2	Vineyard
6	IDT 43	2	Vineyard
7	IDT 46	2	Winery
8	IDT 38	1	Vineyard
9	IDT 39	1	Vineyard
10	IDT 41	1	Vineyard
11	IDT 50	1	Vineyard
12	IDT 51	1	Vineyard
13	IDT 52	1	Vineyard
14	IDT 47	1	Vineyard
15	IDT 48	1	Vineyard
16	IDT 49	1	Vineyard
17	IDT 42	1	Winery
18	IDT 44	1	Winery

Note: The strains were ordered according to their abundance. IDT: inter-delta type.

## Data Availability

The MALDI-TOF spectra for strains examined are held in an in-house database not suited for further dissemination.

## Supplementary Materials

The following supporting information can be downloaded at https://www.mdpi.com/article/10.3390/microorganisms12071299/s1: Table S1: Forty-five commercial wine yeast strains used in this work (kindly provided by Lincoln University Winery).
