# Supplementary material for: Genotyping and Phenotyping of Indigenous Saccharomyces cerevisiae from a New Zealand Organic Winery and Commercial Sources Using Inter-Delta and MALDI-TOF MS Typing
Source: Microorganisms. 2024 Jun 26;12(7):1299. doi: 10.3390/microorganisms12071299 (PMC11278687; doi:10.3390/microorganisms12071299)
Supplement: Supplementary file 1 [file microorganisms-12-01299-s001.zip › microorganisms-3068455-supplementary.pdf]

Table S1. Forty-five commercial wine yeast strains used in this work (kindly provided by Lincoln University Winery).

Yeast strains	Genetic background	IDT	Wine styles[†]
**Commercial strains**			
AWRI Fusion*	*S. cerevisiae* x *S. cariocanus*	30	**White and red wines**
Cepage Cabernet	*S. cerevisiae*	34	**Cabernet Franc as well as Cabernet Sauvignon**
Cepage Chardonnay	*S. cerevisiae* -Strain n° LW05	33	**Chardonnay**
Collection CepagePinot	*S. cerevisiae*	4	**Express the typicity of Pinot Noir**
Cross Evolution	*S. cerevisiae*	28	**White and rosé wines only**
Enartisferm Aroma White	*S. cerevisiae*	11	**White and fruity rosés wines; late harvest sweet wines**
Enoferm AMH™	*S. cerevisiae*	29	**Red wines only**
Enoferm M1	*S. cerevisiae*	17	**White, rosé and dessert wine production**
Fermi champ	*S. cerevisiae*	19	**Ideal yeast for problem fermentation**
Fermicru 4F9*	*S. cerevisiae* -Strain n° 4F9	30	**Aromatic white wines, particularly for ageing on lees**
Fermicru AR2	*S. cerevisiae* -Strain n° L0122	10	**White and rosé wines**
Fermicru Rosé*	*S. cerevisiae* -Strain n°LW10	30	**For rosé wines aroma management**
Fermicru XL	*S. cerevisiae* -Strain n° CECTA 11947	32	**Fruity red wines**
IOC 18-2007*	*S. cerevisiae*	3	**Sparkling wine production; White, rosé, red wines**
Lalvin C	*S. cerevisiae*	23	**White wines, fruit wine and cider production**
Lalvin CLOS	*S. cerevisiae*	26	**Red wines only**
Lalvin CY 3079	*S. cerevisiae*	27	**White wine**
Lalvin DV10*	*S. cerevisiae*	3	**Sparkling, white, rosé and red wine; fruit wine and cider**
Lalvin EC1118*	*S. cerevisiae*	3	**Wide range of application, white, rosé, and red wines**
Lalvin ICV D47	*S. cerevisiae*	35	**Chardonnay and other white varietals**
Lalvin OKAY	*S. cerevisiae*	24	**White, rosé, and red wine**
Lalvin RC212	*S. cerevisiae*	25	**Pinot Noir, red wines**
Lalvin Rhone 2226	*S. cerevisiae*	2	**Red wines**
LalvinQA 23*	*S. cerevisiae*	3	**Fresh, fruit focused whites; high β-glucosidase activity; Riesling**
Levuline BRG	*S. cerevisiae*	15	**White and red wines**

Maurivin AWRI 350	*S. cerevisiae*	12	**White wines; sweet wines**
Maurivin PDM*	*S. cerevisiae*	3	**White and red wines**
Premium Chardonnay	*S. cerevisiae*	13	**White wines**
PREMIUM®PROTIOL	*S. cerevisiae*	3	**White and rosé elegant wines**
Renaissance Allegro	*S. cerevisiae*	22	**An ester-producing yeast for more aromatic modern white wines**
Renaissance Andante	*S. cerevisiae*	8	**Red wines**
Renaissance Brio (Brioso)	*S. cerevisiae*	5	**Red wines**
Renaissance Maestoso	*S. cerevisiae*	7	**Red wines**
Renaissance Vivace	*S. cerevisiae*	20	**White wines**
Rennaissance Ossia	*S. cerevisiae*	21	**An organic yeast for aromatic organic wines; white, red, fruit wines and cider**
Safoeno™ CK	*S. cerevisiae*	10	**White and fruity rosés wines; Vinho Verde type wines**
Sauvignon L3	*S. cerevisiae*	18	**White wines (Sauvignon blanc)**
UCD522	*S. cerevisiae*	14	**Popular-Complex, varietal red wines (or white)**
Velluto Evolution™	*S.cerevisiae/uvarum* (hybrid)	1	**Red wines**
Viniflora Jazz	*S. cerevisiae*	3	**Rosé and Red wines**
Vitilevure Syrah	*S. cerevisiae*	16	**Syrah; red, fruity rosé, and varietal wines**
Zymaflore VL1	*S. cerevisiae*	31	**"*terroir*" white wines (Chardonnays)**
Zymaflore VL3	*S. cerevisiae*	18	**White wines (Sauvignon blanc)**
Zymaflore X5	*S. cerevisiae*	6	**White and rosé wines**
ZYMAFLORE® X16	*S. cerevisiae*	9	**White and rosé wines**
**Wild isolates**			
SV1-28	*S. cerevisiae*	50	
SV2-25	*S. cerevisiae*	36	
SV2-26	*S. cerevisiae*	36	
SV2-27	*S. cerevisiae*	36	
SV3-1	*S. cerevisiae*	36	
SV3-2	*S. cerevisiae*	36	
SV3-3	*S. cerevisiae*	36	

SV3-4	*S. cerevisiae*	36
SV3-5	*S. cerevisiae*	36
SV3-18	*S. cerevisiae*	37
SV2-19	*S. cerevisiae*	37
SV3-20	*S. cerevisiae*	37
SV4-1	*S. cerevisiae*	36
SV4-2	*S. cerevisiae*	36
SV4-3	*S. cerevisiae*	38
SV4-4	*S. cerevisiae*	36
SV4-5	*S. cerevisiae*	36
SV4-6	*S. cerevisiae*	36
SV4-7	*S. cerevisiae*	37
SV4-8	*S. cerevisiae*	39
SV4-9	*S. cerevisiae*	40
SV4-10	*S. cerevisiae*	51
SV4-11	*S. cerevisiae*	3
SV4-12	*S. cerevisiae*	41
SV4-13	*S. cerevisiae*	45
SV4-14	*S. cerevisiae*	40
SV4-15	*S. cerevisiae*	43
SV4-16	*S. cerevisiae*	47
SV4-17	*S. cerevisiae*	52
SV4-18	*S. cerevisiae*	3
SV4-19	*S. cerevisiae*	3
SV4-20	*S. cerevisiae*	45
SV4-21	*S. cerevisiae*	48
SV4-22	*S. cerevisiae*	49
SW2-4	*S. cerevisiae*	36
SW2-5	*S. cerevisiae*	36
SW2-6	*S. cerevisiae*	36

SW3-3	*S. cerevisiae*	36
SW3-5	*S. cerevisiae*	36
SW3-6	*S. cerevisiae*	37
SW3-7	*S. cerevisiae*	36
SW3-8	*S. cerevisiae*	36
SW3-9	*S. cerevisiae*	36
SW4-2	*S. cerevisiae*	36
SW4-3	*S. cerevisiae*	42
SW4-5	*S. cerevisiae*	43
SW4-6	*S. cerevisiae*	36
SW4-7	*S. cerevisiae*	44
SW4-8	*S. cerevisiae*	36
SW4-9	*S. cerevisiae*	36
SW4-10	*S. cerevisiae*	36
SW4-11	*S. cerevisiae*	3
SW4-12	*S. cerevisiae*	3
SW4-13	*S. cerevisiae*	3
SW4-15	*S. cerevisiae*	45
SW4-16	*S. cerevisiae*	45
SW4-17	*S. cerevisiae*	46
SW4-18	*S. cerevisiae*	45
SW4-19	*S. cerevisiae*	46
SW4-20	*S. cerevisiae*	45

* Fermicru_ROSÉ, AWRI_Fusion, Lalvin DV10, Fermicru 4F9, Lalvin EC 1118, Lalvin QA 23, IOC 18-2007, and Maurivin PDM are associated with the Prise de Mousse (PDM) collection of Champagne production (Dunn et al. 2012, Borneman et al. 2016); IDT: inter-delta types; [†]A brief annotation on wine styles based on the manufacturer's recommendations.
